# Estimation of Serum Calcium on the Severity and Mortality in COVID-19 Infections in Sulaymaniyah City, Kurdistan Region of Iraq: A Cross-Sectional Study

**DOI:** 10.3390/clinpract12060103

**Published:** 2022-11-29

**Authors:** Jihad M. Hadi, Shkar M. J. Hassan, Mudhafar M. M. Saeed, Bilal K. Hussein, Banwan M. Ali, Lava E. Muhamad, Ardalan J. Abdullah, Nzar N. Ali, Hawre A. Rahman, Hassan Q. Sofihussein, Jeza M. Abdul Aziz

**Affiliations:** 1 Department of Medical Laboratory of Science, College of Health Sciences, University of Human Development, Kurdistan Regional Government, Sulaimani 46001, Iraq; 2 Pharmacy Department, Sulaimani Technical Institute, Sulaimani Polytechnic University, Kurdistan Regional Government, Sulaimani 46001, Iraq; 3 Emergency Nursing Department, Haibat Sultan Technical Institute, Kurdistan Regional Government, Erbil 46017, Iraq; 4 Business Information Technology Department, Haibat Sultan Technical Institute, Kurdistan Regional Government, Erbil 46017, Iraq; 5 Baxshin Research Center, Baxshin Hospital, Kurdistan Regional Government, Sulaimani 46001, Iraq

**Keywords:** COVID-19, coronavirus, serum calcium, SARS-CoV-2, Sulaymaniyah, Iraq

## Abstract

Background: Coronaviruses (COV) are a large family of viruses that cause infections ranging from the common cold to more serious diseases. Mild to severe respiratory illnesses have been linked to coronavirus disease 2019 (COVID-19), which has been classified as a pandemic disease by the World Health Organization. It has been demonstrated that the severity of COVID-19 is highly positively linked with hypocalcemia. Furthermore, calcium imbalances among other electrolytes are linked to the prognosis of COVID-19. Objectives: This study demonstrates a connection between serum calcium levels and COVID-19 as biomedical indicators of COVID-19 infections in Sulaymaniyah city, Iraq. Methods: A cross-sectional study was conducted at Baxshin Hospital for about two months from February 2022 to April 2022. The work was conducted with a total of 40 patients including 22 males and 18 females. The patients’ ages ranged from 22 to 80 years old. By analyzing a sample from a nasopharyngeal swab and performing real-time reverse transcription-polymerase chain reaction (RT-PCR), all of the patients tested positive as having COVID-19 infection. Serum calcium was determined from the blood samples of the patients in order to evaluate their serum calcium levels. The statistical package for social science (SPSS) was utilized to examine the obtained data. Results: The study revealed a level of calcium between 6.10 and 9.86 mg/dL in male and female patients. The majority of the female patients (61%) displayed low levels of serum calcium, and 33% of the males had a low level of calcium. It can be seen that the highest rate of male patients (66.6%) exhibited a normal level of serum calcium, while 33.3% showed decreased serum calcium. Based on gender and age groups, a statistically significant difference in calcium levels was observed. Conclusions: This study discovered that infection with COVID-19 has some significant laboratory abnormalities, including hypocalcemia, showing that serum calcium might be employed as a prognostic marker in the clinic.

## 1. Introduction

COVID-19 is a contagious disease that can be caused by severe acute respiratory syndrome 2 (SARS-CoV-2) or the new coronavirus (2019-novel) virus, which was called COVID-19 by the World Health Organization (coronavirus disease 19). The first instance was discovered in Wuhan, Hubei Province in China, in December 2019.

Fever, dry cough, exhaustion, breathing difficulties, and a loss of taste and smell are all symptoms of COVID-19, which is a multi-organ dysfunction disease. This virus is rapidly transferred from one person to another by the small drops that form during sneezing and coughing. Although it is most contagious when a person is ill, transmission can happen even before the appearance of the patient’s symptoms. The typical interval between exposure and the start of symptoms is two to fourteen days, with a five-day average. Coughing can cause up to 3000 droplets to form. Despite the fact that these droplets might land on other people and cover adjacent surfaces, some tiny molecules will remain in the atmosphere. Overall, the disease causes mild pneumonia in 79% of cases, more severe symptoms including hypoxia and dyspnea in 15% of cases, and critical and life-threatening situations such as respiratory failure, multi-organ dysfunction, and shock in 15% of cases. A certain number of cases are asymptomatic, but some, such as those in older persons or those with a damaged immune system, are symptomatic [1,2,3].

Coronaviruses are RNA viruses that contain single-stranded positive-sense RNA. This is Coronaviridae’s family member and it is also part of the orthocoronavirinae subfamily. The coronavirus subfamily is split into four genera based on genetic and serological differences that are alpha-, beta-, delta-, and gamma-coronaviruses. The diameter of this spherical coronavirus is 70–120 nm, and the RNA length ranges from 25.5 to 32 kb. There are four various forms of coronavirus in people, birds, and animals. Human coronavirus is caused by the alpha and beta coronaviruses. Moreover, coronaviruses are the most prevalent viruses, with antibodies against them found in 30% to 60% of the Chinese population. Around 80% of the nucleotides in 2019-novel are comparable to those in SARS-CoV. Coronaviruses are made up of different kinds of proteins: the spike protein (S); these proteins help the virus attach to cell receptors and enter the cell. Virus germination is caused by the membrane protein (MP). The virus germinates because of the envelope protein (EP) [4].

A suspect case has previously mentioned symptoms, as well as a history of contact with individuals who have had COVID-19 positive test results. Some people, however, are asymptomatic or even asymptomatic without a fever. A suspected case can be substantiated by a successful molecular test. To make a definite diagnosis, specific molecular studies on respiratory specimens are utilized. A virus might also be discovered in the stool.

Other laboratory tests are frequently non-specific. In most cases, the white cell count is low or normal. There could be lymphopenia; a lymphocyte count of less than 1000 has been associated with serious illness. The current clinical practice recommends measuring IL-6, D-dimer, lactate dehydrogenase (LDH), and transaminases in addition to standard laboratory testing to help identify patients at risk of fatal consequences and those who may benefit from anti-IL-6 immunotherapies using to cilizumab. Surrogate indicators of infection (ferritin, C-reactive protein (CRP)) associated with IL-6 are of growing importance for prognostic usefulness; however, expensive cytokine analysis is not regularly conducted in most laboratories. In most cases, the platelet count is normal or slightly low. CRP and ESR values are frequently increased, although pro-calcitonin levels are normally within normal limits. A high level of pro-calcitonin could indicate bacterial co-infection. Furthermore, the low level of calcium is one of the effects of COVID-19. High levels of GPT/GOT, creatinine, D-dimer, LDH, and prothrombin time are associated with severe diseases [5,6,7]. Napolitano, F. et al. [8] documented a potential serum biomarker for early outcome prediction and clinical severity in hospitalized COVID-19 patients, which is the soluble urokinase receptor. They also discovered that the levels of the soluble urokinase receptor were higher in the mild condition than in a healthy control. However, they were considerably higher with severe illness.

Calcium is one of the most abundant elements in the body, mostly found as hydroxyapatite in the skeleton. Calcium is responsible for much of the structure of teeth and bones, as well as preserving tissue stiffness, flexibility, and strength to facilitate regular physiological movement [9]. The small amount of pool-ionized calcium contained in the circulatory system, multiple tissues, and extracellular fluid mediates dilation and blood artery contraction, clotting of blood, neuronal transmission, hormone secretion, and muscle activity [9,10].

Calcium is obtained from food and dietary supplements, and it is absorbed via the intestinal mucosa through passive diffusion and active transport. Through active transport, the majority of absorption occurs during the lower intake of calcium, while passive diffusion increases the proportion of calcium absorption as raised intake [9,11].

In total, 98% percent of the calcium in the body is stored in the bones, which the body also uses as a calcium source and storage to maintain healthy calcium levels. More than 99% of the calcium in the body is found in the phosphate matrix, calcium hydroxyapatite, and inorganic calcium that is produced in the teeth and bones. [9,11,12]. In contrast to teeth, bone continually resorbs and calcium is deposited into new bone. Bone remodeling is essential to maintain serum calcium levels, change bone size throughout growth, and provide a supply of other minerals [11].

Calcium absorption and ingestion have an inverse relationship. When 200 mg of calcium is taken per day, absorption is approximately 45% but drops to 15% when taking more than 2000 mg per day. [13]. Additionally, age can have an impact on how well dietary calcium is absorbed. In newborns and young children, who require a lot of calcium to develop bone, net dietary calcium absorption can reach as high as 60%, but it drops to around 25% by maturity and keeps declining with age [9]. Blood or plasma can be examined for total calcium levels, and in healthy adults, serum levels typically range from 8.8 to 10.4 mg/dL (2.2 to 2.6 mmol/L). Because of their strict homeostatic control, serum levels do not reflect nutritional status [9,14].

The parathyroid hormone (PTH), vitamin D, phosphorus, and magnesium levels, as well as calcitonin, are all important elements that influence calcium levels. Any of these modulators that are out of balance cause changes in the body and serum calcium levels. Hypercalcemia is linked to an increase in serum (PTH) or vitamin D levels. Hypocalcemia, on the other hand, can be seen in hypoparathyroidism, nephrosis, and pancreatitis [15]. Vitamin D is a fat-soluble vitamin that improves calcium, phosphate, and magnesium absorption, which is essential for appropriate bone formation and maintenance [16]. Furthermore, earlier research has shown a link between vitamin D deficiency and viral processes [17,18]. Moreover, vitamin D plays a physiologic function in maintaining proper innate and adaptive immunity, and it has been demonstrated to reduce pro-inflammatory and boost anti-inflammatory cytokine production, which may benefit COVID-19 patients [19].

One of the major biochemical abnormalities of COVID-19 patients has recently been identified as hypocalcemia. According to numerous studies, COVID-19 individuals exhibit large rates of hypocalcemia and low calcium levels both in hospital emergency rooms and after discharge. In some cases, severe hypocalcemia was also reported. Furthermore, there was a strong association between higher and lower calcium levels of inflammatory parameters and increased disease severity.

Hypocalcemia is commonly seen in severely ill patients and has a greater mortality rate attached to it. Hypocalcemia is common in COVID-2019 individuals, according to studies, and it may be associated with a poor prognostic value in patients with coronavirus disease [20,21]. This work aimed to evaluate the association between calcium levels and COVID-19 in terms of the biochemical markers for SARS-CoV-2 infection in Sulaymaniyah City, Iraq.

## 2. Materials & Methods

### 2.1. Data Collection

In this study, from February 2022 to April 2022, 40 serum samples were obtained from COVID-19-infected individuals in the Baxshin Hospital in Sulaymaniyah province. All of the patients were tested positive for COVID-19 infection via analysis of a sample from a nasopharyngeal swab and performing real-time reverse transcription-polymerase chain reaction (RT-PCR). All the blood samples were taken from patients immediately after being admitted to the hospital and tested positive. In this analysis, several factors, including gender, age, and serum (calcium) were considered by scientific research ethics to compare the biochemical parameters.

### 2.2. Laboratory Testing Determination of Serum (Calcium)

First of all, 40 serum samples were gathered from all the patients to detect the level of calcium utilizing an automated Cobas C-311 (Roche, Germany) device that has a reference range of fewer than 8.5 < normal < 10.5 mg/dL.

## 3. Results

**Figure 1:**
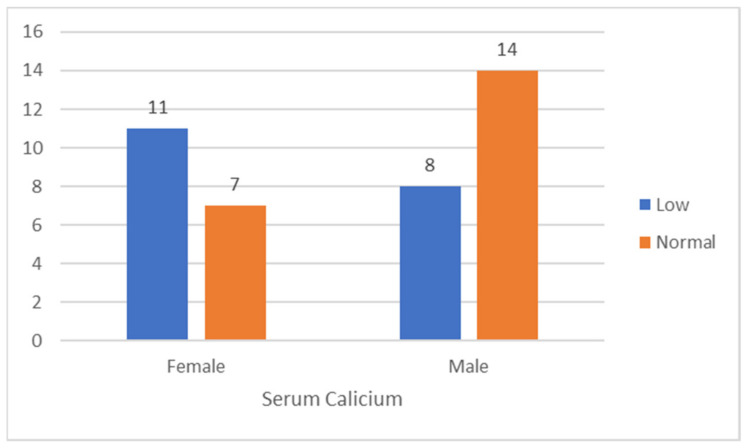
The relationship between gender and calcium level.

In this work, all patients were between the ages of 22 and 80 (mean age of 51.65 years). The COVID-19 patient group included 40 patients with fixed symptoms such as fever, cough, weakness of breath, and numerous organ dysfunctions. The gender distribution of patients in this research was 22 (55%) males and 18 (45%) females. Table 1 and Figure 1 reveal highly significant findings concerning the association between serum calcium levels and SARS-CoV-2 infection. Overall, almost half of patients (52.5%) displayed normal serum calcium, whereas only 47.2% demonstrated hypocalcemia. It can be seen that the highest rate of male patients (66.6%) exhibited a normal level of serum calcium, while 33.3% showed decreased serum calcium. This was also true for female patients, with 38.9% exhibiting normal serum calcium and 61.1% displaying low calcium levels, showing hypocalcemia. It is noteworthy to mention that the calcium level is lower in females compared to male patients, as shown in Figure 1. Table 2 displayed the descriptive statistics according to age and serum calcium.

Moreover, on physical examination, most of the patients had dry mucous membranes, whereas only a few of them had just extra-pulmonic symptoms. Only five patients had underlying diseases, namely, cardiovascular diseases, 2 (5%); diabetes, 2 (5%); and hypertension, 3 (7.5%). The characteristics of COVID-19 gastrointestinal symptoms are subtler than those of COVID-19 respiratory symptoms, making them easy to ignore for all patients. 

**Figure 2:**
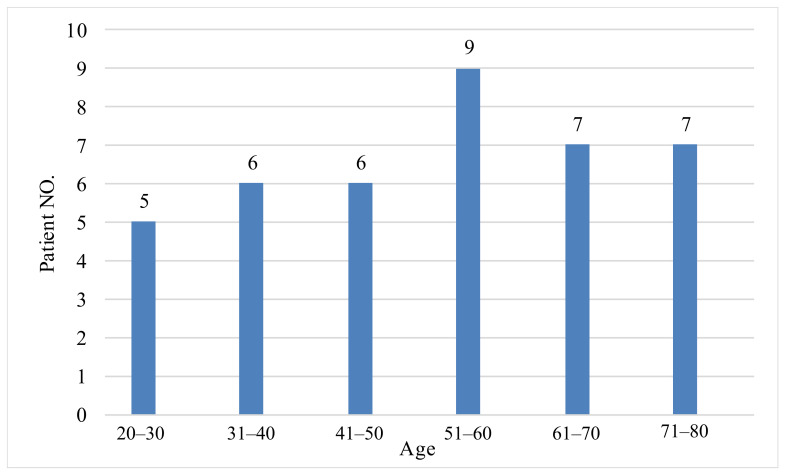
The hypocalcemia according to the age levels.

Figure 2 displays the relationship between hypocalcemia and age levels. It can be observed that hypocalcemia is much higher in the age level of 51–60-year-old patients compared to other levels of age, whereas, for those between 20 and 30 years of age it is relatively lower. On the other hand, there is no significant difference between other age levels, suggesting that age level does not have a significant impact on hypocalcemia. The static analysis for one sample *t*-test for serum calcium, and independent sample test for serum calcium according to gender are shown in Table 3, and Table 4, respectively. 

As shown in the table above, there is a significant difference between the mean sample size of calcium and the standard range (8.5–10.5), and if our *p*-value = 0.00 and less than alpha = 0.05, the null hypotheses can be rejected (H0: null hypotheses: there is no difference between the standard range of calcium and the sample mean calcium), and the alternative hypotheses can be accepted (H1: alternative hypotheses: sample mean calcium is different to that of the standard range of calcium).

The above table illustrates that there is a mean difference between male and female calcium, as Levene’s test *p*-value = 0.02 and is less than alpha 0.05, indicating that male and female calcium are not equal and there is a significant difference between them.

## 4. Discussions

Many viral infections cause serum calcium levels to decrease below 8.8 mg/dL; this condition is known as hypocalcemia. In viral diseases including pneumonia, Ebola, the recent SARS-COV-2 pandemic, and SARS, hypocalcemia is a prevalent anomaly [22,23].

Many studies have documented that most COVID-19 patients had significant hypocalcemia. A result of this study shows hypocalcemia in 45% of patients with COVID-19. In this range of hypocalcemia 55% were female and 45% were male. Other researchers are confident that there is a connection between hypocalcemia and the severity of COVID-19 disease; for instance, Filippo et al. [24] claimed that in COVID-19 patients, hypocalcemia is highly correlated with low vitamin D levels and defective compensatory PTH responses. Their research was carried out in Milan, Italy, at the IRCCS San Raffaele Hospital, a tertiary care facility that is in one of the European centers where the epidemic spread was most active. Their outcomes show that the calcium level was below (2.15 mmol/L), and hypocalcemia was noted in 67.9% of the patients. Moreover, in an extra study, Osman et al. [25] reported that patients with hypocalcemia received more abnormal laboratory results and were in hospital for longer periods of time, and according to their study, hypocalcemia was seen in 60–75% of all age groups. Hypocalcemia has reportedly been linked to the severity of COVID-19 condition. In this investigation, the serum calcium levels of COVID-19 patients and healthy individuals were compared, as well as their association to death and disease severity in COVID-19 patients. According to the findings, calcium levels are much lower in COVID-19 patients than in healthy people.

Although the specific etiology of hypocalcemia in COVID-19 patients with a serious status is unknown, various mechanisms have been proposed: (1) Old age and bad nutrition that results in vitamin D deficiency can both contribute to hypocalcemia. (2) The majority of calcium is linked to plasma albumin, and hypocalcemia can be caused by low serum albumin levels. (3) Proinflammatory cytokines impair parathyroid hormone (PTH) secretion in COVID-19 patients, causing an unbalanced calcium level. (4) Unsaturated fatty acid levels have been shown to be higher in COVID-19 individuals because they can produce hypocalcemia by binding to calcium [26,27].

Despite the fact that people with lower blood calcium levels have a milder illness, numerous studies have demonstrated that their mortality is much greater. Low blood calcium levels related with SARS-CoV-2 infection cannot totally be qualified to the virus’s demand for calcium for life and reproduction because hormonal mechanisms predominantly regulate calcium homeostasis, mainly PTH. Therefore, there could be a connection between inflammation and hypocalcemia [28]. According to some research, cytokines can inhibit calcium receptor expression, causing an unbalanced level of serum calcium. The serum of patients with COVID-19 possessed higher inflammatory markers such as cytokines [29]. In severe disease states, proinflammatory cytokines such as interleukin-6 and interleukin-1 are produced. The relationship between hypocalcemia and more severe infections can be explained by the relationship between serum calcium levels and the immune system, resulting in increased mortality. The findings of this investigation demonstrated that the serum calcium levels of COVID-19 patients are related to disease severity and mortality [30].

## 5. Conclusions

The result of this study indicated that calcium balance is a key contributor to COVID-19 and a biomarker of clinical severity at the onset of symptoms. Hospitalization rates remained higher and illness severity was higher in patients with decreased calcium levels. Calcium is strongly associated with viral multi-organ damage and increased inflammatory cytokines; therefore, serum calcium levels have a critical role in the diagnosis of COVID-19 disease as prognostic biochemical markers. Because serum calcium can be easily measured, for example, during indecisiveness in an emergency, and helps physicians identify COVID-19, initial evaluations could be conceived by assessing serum calcium levels.

## Figures and Tables

**Table 1:** The relation of serum (calcium) with COVID-19 infection.

Calcium	Low	Low in %	Normal	Normal in %	Total	Total in %
Female	11	61.1	7	38.9	18	45
Male	8	33.4	14	66.6	22	55
Grand Total	19	47.5	21	52.5	40	100

Normal range = (8.5 < normal < 10.5) mg/dL.

**Table 2:** Descriptive statistics according to age and serum calcium.

	N	Range	Minimum	Maximum	Mean	S.D.
Age	40	58.00	22.00	80.00	51.6500	16.13016
Serum Calcium	40	3.76	6.10	9.86	8.2400	1.09447

**Table 3:** One sample *t*-test for serum calcium.

	Test Value = 8.5–10.5					
	*t*-Test	Degree of Freedom	Sig. (2-Tailed)	Mean Difference	95% Confidence Interval of the Difference	
					Lower	Upper
Serum Calcium	−7.281	39	0.000	−1.26000	−1.5516	−0.9684

**Table 4:** Independent sample test for serum calcium according to gender.

		Levene’s Test for Equality of Variances		*t*-Test for Equality of Means			
				*t*-Test	Degree of Freedom	Sig. (2-Tailed)	Mean Difference
		F-Test	Sig.				
Serum Calcium	Equal variances assumed	5.892	0.020	2.543	38	0.015	0.82828
	Equal variances not assumed			2.465	30,499	0.020	0.82828

## Data Availability

The primary data for this study is available by request to the corresponding author.
